# Performance, cardioventilatory and perceptual responses to perceived opponent ability during head‐to‐head cycling competition

**DOI:** 10.1113/EP093246

**Published:** 2026-03-17

**Authors:** Guilherme Matta, Andrew Edwards, Bart Roelands, Florentina Hettinga, Philip Hurst

**Affiliations:** ^1^ School of Psychology & Life Sciences Canterbury Christ Church University Canterbury UK; ^2^ Department of Health and Physical Education, Faculty of Liberal Arts and Social Sciences The Education University of Hong Kong Hong Kong Hong Kong; ^3^ Human Physiology and Sports Physiotherapy research group (MFYS) Vrije Universiteit Brussel (VUB) Brussels Belgium; ^4^ Laboratory of Sports and Nutrition Research Riga Stradiņš University Riga Latvia; ^5^ Department of Human Movement Sciences, Faculty of Behavioural and Human Movement Sciences Vrije Universiteit Amsterdam The Netherlands

**Keywords:** competitive behaviour, deception, endurance exercise, exercise regulation, expectation

## Abstract

We aimed to investigate whether cycling competition influences performance and pacing compared to an individual time trial. The second aim was to determine whether performance is further influenced by deceptive or accurate augmented feedback, delivered as an opponent riding at 2% higher power outputs than a previous competitive time trial. Ventilatory, metabolic and perceptual responses were examined. Twelve male cyclists completed six laboratory sessions: (1) incremental test and familiarisation; (2) baseline 20‐min individual time trial; (3–4) time trial against a virtual opponent replicating the baseline performance (Competition_BSL_); and (5–6) augmented feedback trials against an opponent riding at 2% higher power outputs than Competition_BSL_, presented as either Accurate (participants informed of the 2% increase) or Deception (told the opponent may have received an ergogenic aid). Power output, pacing, gas exchange, blood lactate, heart rate, ratings of perceived exertion (RPE), and motivation were recorded. Power output was higher only during Accurate (*P = *0.032) compared to Baseline, accompanied by higher perceived exertion and ${\dot{V}}_{CO_{2}}$. Relative to Baseline, no differences were observed in ${\dot{V}}_{O_{2}}$, ${\dot{V}}_{E}$, heart rate, RER, post‐exercise blood lactate concentration, or motivation in any condition. Linear mixed‐effects modelling indicated a curvilinear pacing across time, with no differences in pacing curve between conditions. Our findings may indicate that competitive framing, rather than the mere presence of an opponent, influenced performance during fixed‐duration cycling time trial.

## Abstract (highlights)

- **What is the central question of this study?**
  Does head‐to‐head cycling competition improve performance relative to an individual time trial, and how do perceptions of opponent ability through accurate versus deceptive augmented feedback affect performance, cardioventilarory and perceptual responses?
- **What is the main finding and its importance?**
  Head‐to‐head competition did not consistently enhance performance relative to an individual time trial. Performance improved when cyclists were informed that their opponent was riding at 2% higher power outputs (Accurate condition), whereas performance returned to baseline levels when the opponent was perceived as potentially advantaged (Deception). Performance changes were accompanied by higher perceived exertion, likely indicating greater voluntary task engagement and perceived goal attainability.

## 1 INTRODUCTION

The dynamics of racing and competition have attracted scientific interest for over a century (Triplett, 1898; Wilmore, 1968). The seminal work of Triplett (1898) was the first to show that cyclists improved performance in the presence of an opponent in comparison to when riding alone. Several decades later, Wilmore (1968) reported comparable ventilatory and cardiovascular responses during competitive and individual time trials, highlighting the importance of psychological constructs such as motivation in shaping exercise behaviour. Since then, research (Davies et al., 2016; Hettinga et al., 2017; Hibbert et al., 2018; Konings et al., 2016; Williams et al., 2014) has consistently shown that the presence of opponents can improve endurance exercise performance, partly through changes in pacing and motivation.

To further explore such athlete–environment interactions, studies have manipulated athletes’ expectations about their opponents. In such designs, participants unknowingly race against a competitor set to perform at power outputs above a previous individual baseline performance – referred to as augmented feedback. For example, Ansdell et al. (2018) found that competing against an opponent covertly set 2% above an individual time trial, while believing the opponent matched their previous effort, improved 4‐km performance from 367 ± 15 s to 361 ± 17 s; it also increased post‐exercise blood lactate concentration ([La^−^]), despite similar rating of perceived exertion (RPE). Interestingly, when participants were informed about the nature of the opponent (i.e., accurate condition), performance, RPE and [La^−^] returned to baseline values. Similarly, Ducrocq et al. (2017) found ∼5% improvements in performance during 5‐km time trials when participants were instructed to follow an opponent riding 2% faster than baseline, although deceptively informed the opponent replicated their previous performance. Performance improvements were associated with higher ${\dot{V}}_{O_{2}}$, ${\dot{V}}_{CO_{2}}$ and ${\dot{V}}_{E}$, suggesting an increased ventilatory response during competition. Other studies reported similar findings (Jones et al., 2016a; Stone et al., 2012; Williams et al., 2016; Williams, Massey et al., 2015), suggesting that augmented feedback might enhance performance in comparison to an individual time trial. However, these previous studies manipulated opponents’ performance in relation to participants’ individual time trial. Although informative, such a strategy may elicit performance changes attributable to social facilitation (Edwards et al., 2018), rather than the deceptive intervention per se. To more precisely isolate the effects of augmented feedback, it is first necessary to establish the performance changes that would occur when moving from an individual to a competitive time trial before introducing further opponent manipulations.

The perceived challenge posed by an opponent can also influence both exercise regulation (e.g., pacing) and motivation (Hibbert et al., 2018; Parton & Neumann, 2019; Strauss, 2002). Some studies suggest that competing against a seemingly superior opponent can impair performance and reduce motivation (Ducrocq et al., 2017; Parton & Neumann, 2019). However, it remains unknown whether these effects extend to situations in which an opponent is perceived to have an advantage through the use of an ergogenic aid. This question is particularly relevant given the widespread use of nutritional interventions to improve performance (Burke, 2021) and could offer new insights into how the perception of opponents influences competitive behaviour.

The first aim of this study was to investigate whether simulated head‐to‐head competition affects cycling performance and pacing in comparison to an individual time trial. The second aim of this study was to assess whether performance is further affected by the provision of both deceptive and accurate augmented feedback in the form of an opponent riding at 2% higher power outputs than a previous competitive time trial. Within those aims, we aimed to investigate the cardioventilatory, metabolic and perceptual responses between baseline and the subsequent competitive time trials.

## 2 METHODS

### 2.1 Ethical approval

The lead author's institutional human research committee approved the study in compliance with the *Declaration of Helsinki* (ref.: ETH2021‐0364), except for registration in a database; and all participants provided written informed consent to participation.

### 2.2 Participants (*n* = 12)

Fourteen participants were initially recruited, but two withdrew due to injury unrelated to the study protocol. Therefore, 12 trained male cyclists composed the final sample (Table 1). Eligibility criteria stipulated participants were 18–55 years old, performing >6 h of cycling per week, regularly completing self‐paced time trials for training, performance assessment or competition, free of any neuromuscular injury and had not experienced upper‐respiratory tract illness symptoms in the 2 months preceding participation.

**TABLE 1:** Participants' characteristics (*n* = 12) and preliminary test results.

Age (years)	38 ± 8
Height (cm)	182 ± 7
Body mass (kg)	74.9 ± 10.3
${\dot{V}}_{O_{2}\max}$ (mL kg^−1^ min^−1^)	58.6 ± 7.2
${\dot{V}}_{O_{2}\max}$ (L min^−1^)	4.43 ± 0.41
${\dot{V}}_{\mathrm{Epeak}}$ (L min^−1^)	179 ± 14
RER_peak_	1.22 ± 0.06
*Ẇ*_max_ (W)	400 ± 41
*Ẇ*_max_ (W kg^−1^)	5.40 ± 0.63
Maximal heart rate (beats min^−1^)	180 ± 14

Data are means ± SD. RER_peak_, peak respiratory exchange ratio; ${\dot{V}}_{\mathrm{Epeak}}$, peak min ventilation; ${\dot{V}}_{O_{2}\max}$, maximal oxygen uptake; *Ẇ*_max_, maximal work rate during incremental test.

### 2.3 Experimental design

**FIGURE 1:**
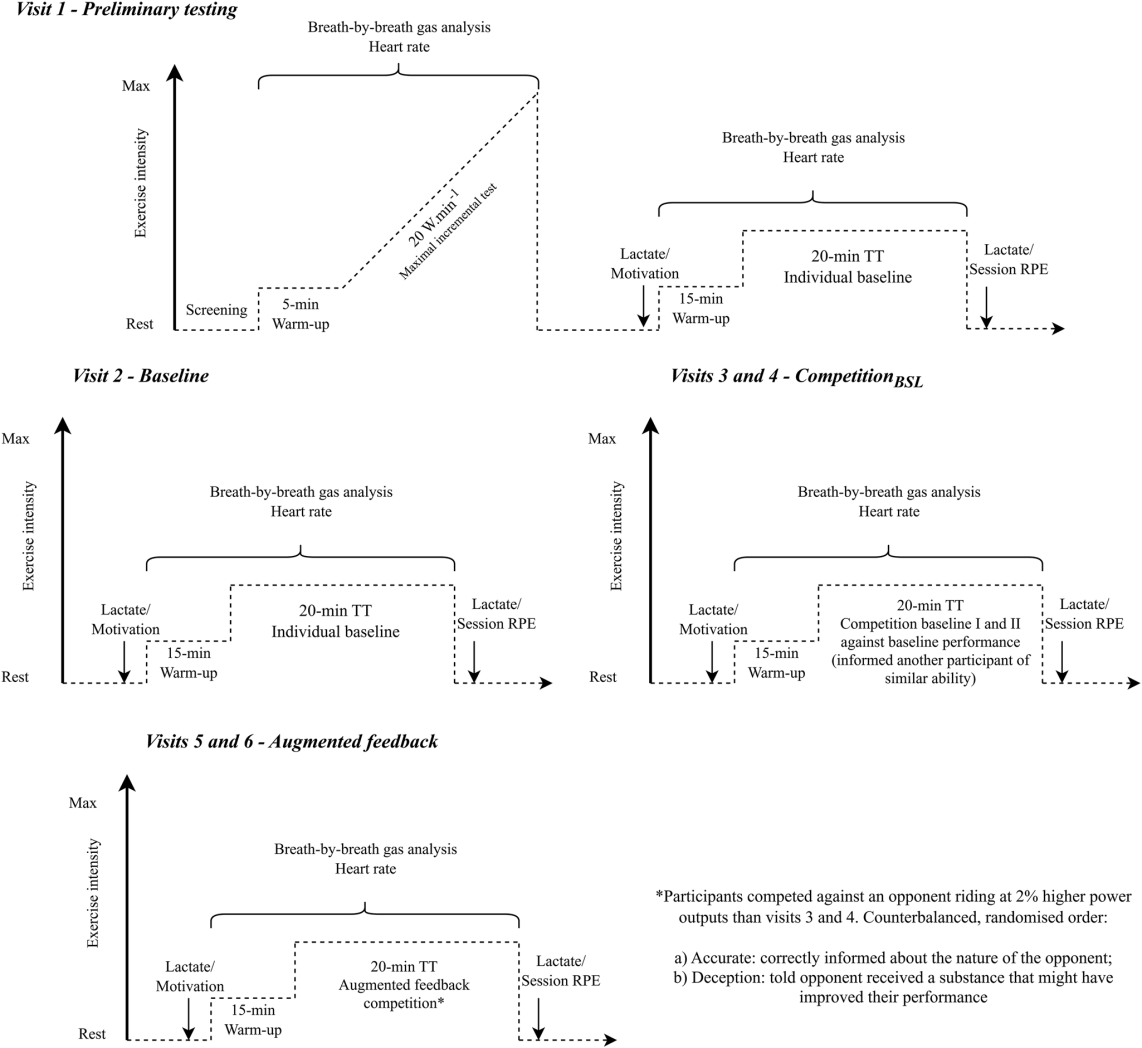
Schematic overview of the study design.

A within‐subject, repeated‐measures experimental design was employed, with participants attending the laboratory on six occasions at the same time of day (±2 h), each separated by at least 48 h but no more than 72 h (Figure 1). In the first session, participants performed an incremental test and were familiarised with the 20‐min time trial protocol. The second session included an individual time trial, whereas visits 3–6 consisted of head‐to‐head competitions. Time trials started with a 15‐min warm‐up prescribed in relation to the RPE scale (Borg, 1982), followed by a 20‐min time trial that differed only in the information provided to participants regarding their opponents. We adopted a deceptive intervention, with participants informed that the study aimed to investigate the effects of exogenous ketones on competitive cycling performance, and that on one occasion they would compete against another participant who had ingested either a ketone drink or a placebo before the time trial (see ‘Experimental procedures’). At the end of the study, participants were fully debriefed about its true purpose and given the option to withdraw their data.

### 2.4 Preliminary testing

First, participants’ height and body mass were measured. Subsequently, they performed a maximal incremental test on an electromagnetically braked cycle ergometer (Velotron, RacerMate, Seattle, WA, USA). Participants adjusted the bike dimensions before the start (replicated throughout the subsequent visits) and warmed up for 5 min at 80 W. The test started at 100 W, followed by increases of 20 W every minute until voluntary exhaustion or inability to maintain a cadence above 60 rev min^−1^ despite verbal encouragement. Participants were instructed to adopt a self‐selected cadence but to avoid abrupt changes throughout the test. Gaseous exchange was collected continuously using a breath‐by‐breath metabolic analyser (Vyntus CPX, Jaeger‐CareFusion, Höchberg, Germany) and ${\dot{V}}_{O_{2}\max}$ was defined as the highest 30‐s mean. Before the test, the gas analyser was calibrated as per the manufacturer's instructions, using standard gases of known concentrations. Power output and heart rate (Polar H7, Polar Electro, Kempele, Finland) were recorded throughout the test, and the maximal power output (*Ẇ*_max_) was defined as the last 5‐s mean.

Following 30 min of recovery, participants were familiarised with the 20‐min time trial protocol (including the warm‐up, described below), of which they were instructed to achieve the highest possible power output.

### 2.5 Baseline and head‐to‐head competitions

Participants completed five 20‐min time trials: one individual baseline, two competitive baselines, and two experimental competitive trials, all performed on a flat virtual course projected on a screen that depicted them as graphical avatars. Before the start of each time trial, participants completed a 15‐min warm‐up, following the protocol described by Bossi et al. (2020). It consisted of 5 min at an RPE intensity corresponding to 11 (light), followed by three 1‐min high‐intensity intervals at RPE 16 (between hard and very hard), interspersed by recovery periods of 2 min at RPE 9 (very light), and a final 3 min at RPE 9. Participants could adjust gear ratio and cadence to achieve the prescribed RPE and were given a maximum of 3 min after the warm‐up to drink water and get ready for the time trial.

Baseline (visit 2) consisted of an individual 20‐min time trial, during which participants were requested to achieve the highest possible power output. During sessions 3 and 4, they competed against a virtual opponent replicating their baseline overall performance and pacing. To induce a sense of competition, participants were informed that the opponent represented the performance of another participant of a similar level. The average performance across sessions 3 and 4 was defined as a competitive baseline (Competition_BSL_) and was subsequently used to establish the opponent's performance in experimental sessions 5 and 6 (see below). The average of sessions 3 and 4 was used to provide a more reliable measurement of performance by reducing random error and within‐subject variability, in line with previous recommendations (Hecksteden et al., 2018).

Sessions 5 and 6 consisted of augmented feedback time trials, presented in a randomised, counterbalanced order as (a) Accurate and (b) Deception. In both conditions, participants competed against an opponent representing the performance and pacing achieved during Competition_BSL_, but with power outputs increased by 2%. In the Accurate condition, participants were informed about the 2% increase and that closely following the opponent would improve their performance. In the Deception condition, however, participants were informed that they would compete against another participant of the study of a similar level, but who might have received an exogenous ketone drink.

### 2.6 Experimental procedures

Participants completed all time trials on the same electromagnetically braked cycle ergometer (Velotron, RacerMate), which has been shown to accurately measure power output (Abbiss et al., 2009). Before each time trial, the ergometer was calibrated using the Accuwatt™ verification procedure. Briefly, this involved accelerating the flywheel to 23 miles h^−1^ and allowing it to decelerate, with the software assessing the rate of decline in angular velocity to confirm that the system was within factory calibration settings.

A 20‐min time trial was chosen for different reasons. First, it provides a reliable measurement of performance and pacing (MacInnis et al., 2019; Matta et al., 2022; Nimmerichter et al., 2010). Second, although distance‐based trials may better reflect real‐world competition, their translation to an indoor ergometer requires converting power into speed, which is influenced by factors such as aerodynamics, body size and environmental conditions, not accounted for by the ergometer's power–speed calculations (Currell & Jeukendrup, 2008). Third, cyclists typically perform time‐based high‐intensity interval training (Bucheit & Laursen, 2013) and performance assessment tests (Leo et al., 2022), making this format familiar. Finally, time‐based trials standardise exercise duration across participants and, therefore, offer greater experimental control (Currell & Jeukendrup, 2008).

The ketone narrative was selected as exogenous ketone supplementation is a topical and plausible ergogenic aid in cycling (Evans et al., 2022). Importantly, the scientific evidence supporting its performance benefits remains equivocal, reducing the likelihood that participants held strong preconceptions that might bias behaviour. Presenting the opponent as potentially superior provided a realistic and credible rationale for the experimental manipulation, while concealing the true purpose of the study. The 2% increase in power output was based on previous studies employing similar designs (Davies et al., 2016; Jones et al., 2013; Williams et al., 2014), and represents the smallest worthwhile change in performance (Hopkins et al., 1999), thereby minimising the likelihood of detection.

Participants were instructed to maintain their regular training but to refrain from high‐intensity exercise 24 h before each session, and to prepare as they would for competition. They were also instructed to abstain from caffeine for 3 h before each visit and to replicate their diet as closely as possible over the preceding 24 h. Every visit was performed in a laboratory‐controlled environment (18–19°C, 40% humidity), and a cooling fan was positioned behind the participants. During each time trial, all feedback was hidden (i.e., power output, cadence, heart rate, speed and distance), apart from time elapsed.

### 2.7 Outcome measures

Before the start of each time trial, participants completed a motivation questionnaire (Matthews et al., 2001), composed of 14 statements scored on a 5‐point Likert scale (0 = not at all to 4 = extremely) that assessed intrinsic and success motivation.

During all time trials, power output and cadence were measured continuously by the ergometer. Each time trial file was extracted from the Velotron 3D (2008) software in a Flexible and Interoperable Data Transfer (FIT) format and subsequently analysed using training‐analysis software (TrainingPeaks WKO+ v.3.0, Peaksware, Louisville, CO, USA). Heart rate (Polar H7), pulmonary ventilation and gas exchange (Vyntus CPX, Jaeger‐CareFusion) were measured continuously. Respiratory data were smoothed into 10‐s intervals, and the mean ${\dot{V}}_{O_{2}}$, ${\dot{V}}_{CO_{2}}$, ${\dot{V}}_{E}$ and RER were recorded for each time trial.

Before and after each time trial (<2 min), blood lactate concentration (Pre and Post [La^−^]; 3 µL) was collected from a fingertip of the participants’ right hand and immediately analysed with a test strip by electrochemical method (LactatePro 2 Lactate Meter, Arkray Inc., Kyoto, Japan). After approximately 20 min, the session RPE (sRPE) was recorded on a 0 (rest) to 10 (maximal) scale (Foster et al., 2001).

### 2.8 Data analysis

All data are reported as means ± standard deviation. Differences between conditions for mean absolute power output, cadence, heart rate, Pre and Post [La^−^], ${\dot{V}}_{O_{2}}$, ${\dot{V}}_{CO_{2}}$, ${\dot{V}}_{E}$, RER, sRPE, and intrinsic and success motivation were analysed using linear mixed‐effects models. In each model, condition was included as a fixed effect and participant was included as a random intercept to account for the repeated‐measures design and within‐participant dependence of observations. Although the primary comparisons of interest involved baseline versus competitive conditions, all pairwise contrasts between the four conditions were evaluated using Tukey‐adjusted estimated marginal means. Degrees of freedom for fixed effects were estimated using the Kenward–Roger approximation.

To analyse pacing, mean power output from each 2‐min segment was normalised as a percentage of the mean power output achieved across the entire 20‐min trial for each participant, hereafter referred to as normalised power output (%). This approach characterises pacing independently of differences in absolute power output between participants and conditions (Davies et al., 2016; Thomas et al., 2012). Pacing was then analysed using linear mixed‐effects models, following the procedures described by Bossi et al. (2025). A hierarchical modelling strategy was adopted to determine the most appropriate representation of the pacing curve. Four nested models of increasing complexity were fitted: (i) an intercept‐only model, (ii) a linear time model, (iii) a quadratic time model, and (iv) a quadratic time model including condition, condition × time, and condition × time^2^ interactions. Time was represented as time trial segment (2‐min intervals) and was mean‐centred prior to modelling to improve interpretability and reduce collinearity between linear and quadratic terms. All models included a random intercept for participant ID to account for repeated measures. Random slopes were not included as the normalisation procedure constrains each participant's mean normalised power output to 100%, resulting in negligible between‐participant variance. Models were fitted using maximum likelihood estimation. Nested models were compared using likelihood‐ratio tests and Akaike's information criterion (AIC), with lower AIC values indicating better relative model fit.

All analyses were performed using R version 4.5.2 (R Foundation for Statistical Computing, Vienna, Austria) with linear mixed‐effects models fitted using the *lme4* package, degrees of freedom estimated via *lmerTest*, and pairwise contrasts computed using *emmeans*. Statistical significance was set at *P* ≤ 0.05.

## 3 RESULTS

All descriptive results are presented in Table 2.

**TABLE 2:** Performance, cardioventilatory, metabolic and perceptual responses of each condition (*n* = 12).

	Baseline	Competition_BSL_	Deception	Accurate
Power output (W)	276 ± 38	283 ± 38	280 ± 36	284 ± 40^*^
Cadence (rev min^−1^)	97 ± 8	98 ± 7	100 ± 8	98 ± 7
Heart rate (beats min^−1^)	169 ± 12	170 ± 13	169 ± 13	168 ± 12
Pre [La^−^] (mmol L^−^^1^)	1.72 ± 0.50	1.85 ± 0.40	1.98 ± 0.48	1.82 ± 0.89
Post [La^−^] (mmol L^−^^1^)	12.00 ± 3.66	13.10 ± 4.13	11.75 ± 4.07	12.37 ± 5.08
${\dot{V}}_{E}$ (L min^−1^)	128 ± 24	132 ± 20	133 ± 18	133 ± 22
${\dot{V}}_{O_{2}}$ (L min^−1^)	3.73 ± 0.40	3.83 ± 0.37	3.82 ± 0.33	3.81 ± 0.42
${\dot{V}}_{CO_{2}}$ (L min^−1^)	3.73 ± 0.35	3.85 ± 0.34	3.88 ± 0.29^*^	3.89 ± 0.40*
RER	1.00 ± 0.02	1.01 ± 0.03	1.01 ± 0.03	1.02 ± 0.03
sRPE (AU)	8.3 ± 1.3	8.6 ± 0.9	8.7 ± 0.9	9.2 ± 0.6^*^
Intrinsic motivation (AU)	23.8 ± 3.6	24.5 ± 3.6	24.4 ± 3.9	23.8 ± 3.9
Success motivation (AU)	20.9 ± 3.0	20.8 ± 3.7	21.8 ± 3.9	21.6 ± 3.6

Data are means ± SD. *Different from Baseline. AU, arbitrary units; [La^−^], blood lactate concentration; RER, respiratory exchange ratio; sRPE, session ratings of perceived exertion; ${\dot{V}}_{CO_{2}}$, carbon dioxide production; ${\dot{V}}_{E}$, min ventilation; ${\dot{V}}_{O_{2}}$, oxygen consumption.

### 3.1 Performance outcomes

**FIGURE 2:**
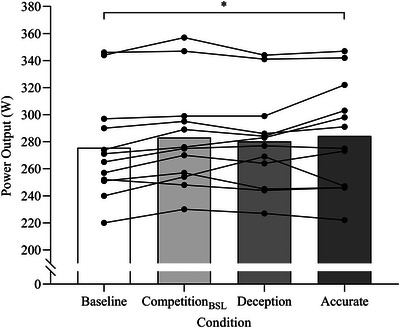
Individual values for mean power output (W) across conditions. *Significant difference (*P* = 0.032). Standard deviation error bars are not shown to avoid visual clutter; standard deviation values for each condition are reported in Table 2.

We found a significant main effect of condition on power output (Figure 2; *F*_3,33_ = 3.24, *P* = 0.034), and Tukey‐adjusted pairwise comparisons indicated that power output was higher in the Accurate condition compared with Baseline (Table 3; *P* = 0.032). We found no main effect of condition on cadence (*F*_3,33 _= 1.76, *P* = 0.174).

**TABLE 3:** Tukey‐adjusted pairwise comparisons for mean absolute power output (W) between each condition.

Contrast	Mean difference (W)	95% CI (W)	Adjusted *P*‐value
Competition_BSL_–Baseline	7	−1 to 15	0.094
Deception–Baseline	5	−4 to 3	0.422
Accurate–Baseline	9	1 to 17	0.032^*^
Deception–Competition_BSL_	−3	−11 to 6	0.820
Accurate–Competition_BSL_	2	−7 to 10	0.962
Accurate–Deception	4	−4 to 12	0.537

* Significant difference.

### 3.2 Cardioventilatory and metabolic outcomes

We found no main effect of condition on heart rate (*F*_3,33_ = 0.79, *P* = 0.508), Pre [La^−^] (*F*_3,33_ = 0.58, *P* = 0.634), Post [La^−^] (*F*_3,33 _= 1.58, *P* = 0.213), ${\dot{V}}_{E}$ (*F*_3,33_ = 2.19, *P* = 0.108), ${\dot{V}}_{O_{2}}$ (*F*_3,33_ = 2.51, *P* = 0.076) and RER (*F*_3,33_ = 2.01, *P* = 0.132). However, there was a main effect of condition on ${\dot{V}}_{CO_{2}}$ (*F*_3,33_ = 4.49, *P* = 0.010) and Tukey‐adjusted pairwise comparisons indicated that in comparison to Baseline, ${\dot{V}}_{CO_{2}}$ was higher in Deception (∆ = 0.150 L min^−1^, 95% CI = 0.016 to 0.284; *P* = 0.024) and Accurate (∆ = 0.162 L min^−1^, 95% CI = 0.028 to 0.296; *P* = 0.013).

### 3.3 Perceptual outcomes

There was a main effect of condition on sRPE (*F*_3,33 _= 3.38, *P* = 0.030), and Tukey‐adjusted pairwise comparisons indicated that sRPE was higher in Accurate compared with Baseline (∆ = 0.92 A.U, 95%CI = 0.13 to 1.71; *P* = 0.018).

### 3.4 Pacing

**FIGURE 3:**
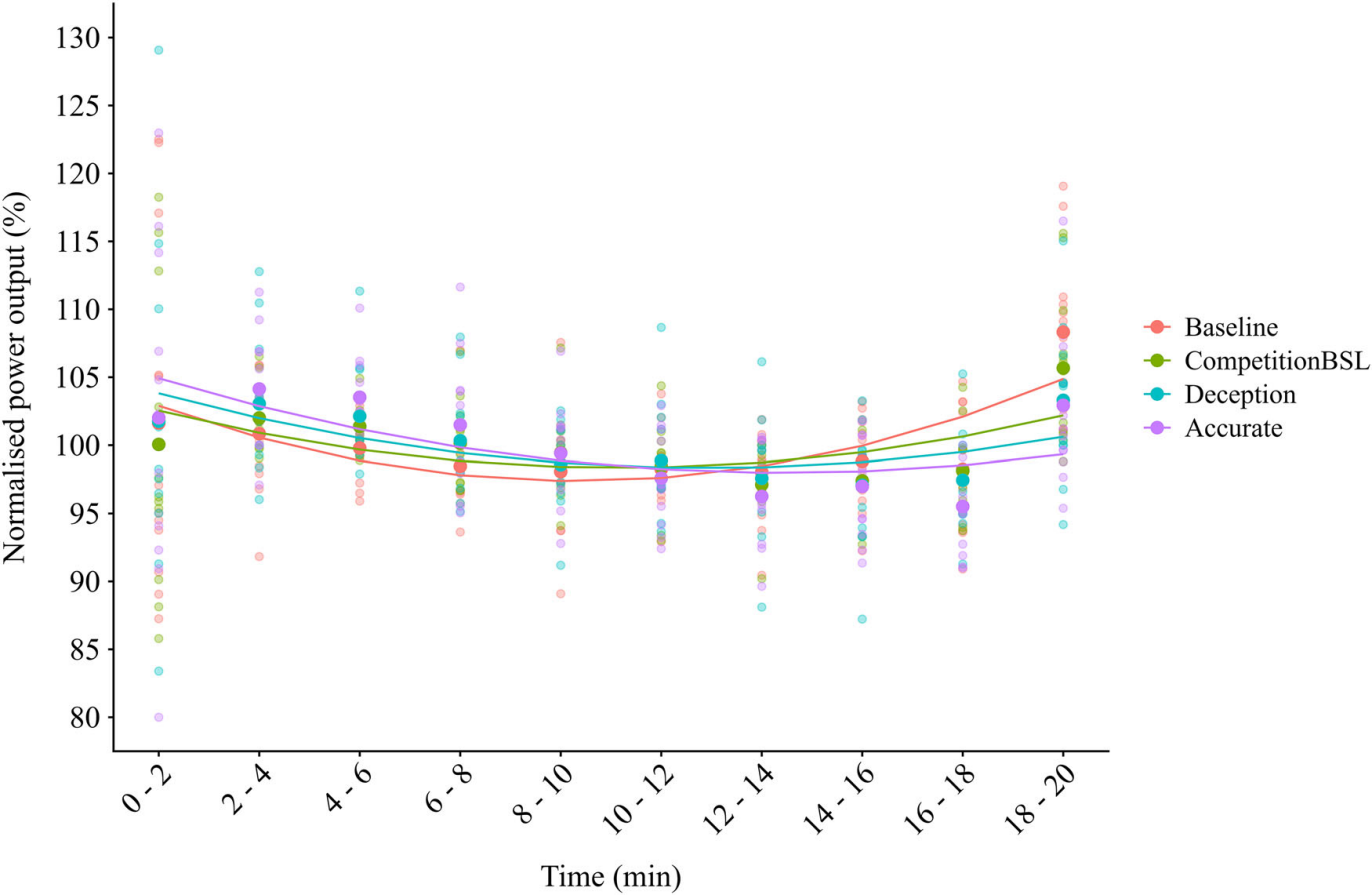
Pacing profile during the 20‐min time trials. Mean power output from each 2‐min segment is expressed as a percentage of each participant's mean power output across the time trial (normalised power output, %). Dots represent individual participant values for each segment. Lines represent model‐estimated pacing curves from the quadratic linear mixed‐effects model. Pacing followed a curvilinear pattern across time, with no differences in pacing profile between conditions.

Model comparison indicated that pacing was not adequately described by an intercept‐only model. Adding a linear time term significantly improved model fit compared with the intercept‐only model (χ^2^(1) = 4.80, *P* = 0.028). Model fit improved further with the inclusion of a quadratic time term, with the quadratic model providing a significantly better fit than the linear model (χ^2^(1) = 39.52, *P* < 0.001; ΔAIC = −37.5), indicating a curvilinear pacing across the time trials. Including condition and condition × time interaction terms did not significantly improve model fit compared with the quadratic time model (χ^2^(9) = 16.47, *P* = 0.058; ΔAIC = +1.5). Therefore, although pacing followed a curvilinear profile across time, the pacing curve did not differ between conditions (Figure 3).

## 4 DISCUSSION

Our findings indicate that the effects of competition on performance are contingent on how the competitive setting is framed. In comparison to Baseline, mean power output was higher in the Accurate condition, where participants competed against an opponent riding at 2% higher power output and were correctly informed of this. In contrast, no differences were observed between Baseline and Deception or Competition_BSL_ conditions. These results suggest that the presence of an opponent itself may not induce performance improvements; instead, how the competition is framed likely shapes how participants approach cycling fixed‐duration time trials.

### 4.1 Augmented feedback – Deception and Accurate

Augmented feedback in the form of an opponent riding at higher power outputs than what cyclists are led to believe has been shown to improve performance (Ducrocq et al., 2017; Jones et al., 2016a; Stone et al., 2012; Williams et al., 2016; Williams, Massey et al., 2015). However, previous studies manipulated opponents’ performance in relation to an individual time trial. Despite providing important insights into the dynamics of head‐to‐head competitions, those observed performance improvements may not accurately represent a true change to baseline, as the mere presence of an opponent might affect performance through social facilitation (Edwards et al., 2018; Triplett, 1898).

During both augmented feedback conditions in our study (i.e., Accurate and Deception), the opponent replicated the participants’ competitive performance, rather than that of an individual time trial. Compared with Baseline, mean power output was higher in the Accurate condition, whereas no significant differences were observed in the Deception condition. Similar to Ducrocq et al. (2017), during the Accurate condition, participants were informed that closely following the opponent would induce performance improvements. However, during Deception, they were instructed to win the race. It has been previously shown that goal orientation (Crivoi et al., 2022; Hibbert et al., 2018; Rhoden et al., 2015) and exercise expectation (Viana et al., 2025) influence performance. It is plausible that the goal of winning (Deception) in comparison to the goal of improving their best performance (Accurate) influenced how participants approached each time trial. For example, Crivoi et al. (2022) showed that racing against a faster opponent reduced self‐efficacy, despite no changes in performance. Although speculative, the more self‐referenced goal in the Accurate condition may have facilitated performance, whereas the winning‐oriented goal in the Deception condition may have attenuated it. Given our relatively small sample size, future research with larger samples is warranted to determine the robustness of these effects.

During Accurate, performance improvements were accompanied by higher sRPE and ${\dot{V}}_{CO_{2}}$ in comparison to Baseline. Similarly, Jones et al. (2016a) and Jones et al. (2016b) found higher RPE during 16.1‐km time trials when participants competed against an opponent riding 2% faster than baseline. Ducrocq et al. (2017) also reported greater RPE during a 5‐km time trial when participants followed a virtual pacer set at 5% above baseline. However, several studies reported no changes in RPE during augmented feedback conditions in comparison to baseline (Ansdell et al., 2018; Crivoi et al., 2022; Konings et al., 2018; Shei et al., 2016; Stone et al., 2017; Williams et al., 2014). This might suggest that RPE is context dependent and shaped by how the task is framed and interpreted by the athlete. In our study, the higher sRPE during Accurate was accompanied by improved performance and increased ${\dot{V}}_{CO_{2}}$, likely indicating greater voluntary task engagement. In contrast, during Deception, athletes may have regulated effort more conservatively, resulting in unchanged sRPE despite similar demands. The higher ${\dot{V}}_{CO_{2}}$ observed during Accurate and Deception is consistent with findings from Stone et al. (2012) and Corbett et al. (2012), and could indicate subtle alterations in CO_2_ kinetics or acid–base regulation during exercise. However, this should be interpreted with caution, as ${\dot{V}}_{O_{2}}$, ${\dot{V}}_{E}$, RER and Post [La^−^] remained unchanged. Therefore, ${\dot{V}}_{CO_{2}}$ differences may reflect small changes in buffering or ventilatory efficiency rather than a meaningful increase in metabolic demand. Intrinsic and success motivation did not differ between Accurate and Baseline, contrasting with prior suggestions that performance improvements are associated with increased motivation (Jones et al., 2013; Williams et al., 2014). These findings suggest that Accurate augmented feedback may enhance performance possibly via central mechanisms related to goal orientation (Crivoi et al., 2022) and not necessarily through motivation.

### 4.2 Competition_BSL_ versus baseline

Consistent with Ducrocq et al. (2017), Konings et al. (2020) and Wood et al. (2020), no difference in performance was observed when participants competed against an opponent replicating their Baseline performance; however, this contrasts with the wider literature (Davies et al., 2016; Jones et al., 2016a; Stone et al., 2012; Williams et al., 2016; Williams, Massey et al., 2015). For example, Corbett et al. (2012), Williams, Jones et al. (2015) and Konings et al. (2016) reported performance improvements during 2‐, 16.1‐ and 4‐km cycling time trials, respectively, when participants unknowingly competed against an opponent replicating their individual performance. One possible explanation for these discrepancies may relate to goal orientation. During Competition_BSL_, participants were instructed to race the opponent, which may have promoted a winning‐oriented goal rather than performance improvement, consistent with decision‐making frameworks in endurance exercise (Renfree et al., 2014). In such contexts, winning can be achieved without increasing overall power output or speed, as cyclists only need to finish ahead of their opponent. Nevertheless, the wide confidence intervals between Competition_BSL_ and Baseline (Table 3) indicate some uncertainty in the magnitude of the effect. While no significant difference was detected, the absence of clear effects should not be interpreted as definitive evidence that competing against a matched opponent has no influence on performance.

No differences were observed between Competition_BSL_ and Baseline for any ventilatory, metabolic and perceptual responses. Previous studies have similarly reported that competition does not necessarily alter physiological and perceptual responses, even when performance is improved. For example, Ducrocq et al. (2017) reported no differences in ${\dot{V}}_{O_{2}}$, ${\dot{V}}_{CO_{2}}$, ${\dot{V}}_{E}$, RER, Post [La^−^] and heart rate when participants competed against an opponent replicating their Baseline performance. Konings et al. (2016) and Williams et al. (2016) reported that although cycling performance improved during competition, RPE was rated similarly to an individual time trial. It may be that perceptual responses such as RPE may not always reflect subtle changes in internal load that can emerge in competitive settings. In our study, motivation was also not different between Competition_BSL_ and Baseline. This aligns with evidence suggesting that motivation does not always increase in response to competitive contexts (Shei et al., 2016), although subtle changes may be difficult to detect using global assessments of motivational state. The lack of differences in ventilatory, metabolic and perceptual responses suggests that the competitive setting did not induce greater physiological or perceptual demands than Baseline, which may be consistent with the winning‐oriented task framing.

### 4.3 Pacing

To analyse pacing, we modelled normalised power output across the 20‐min time trials using linear mixed‐effects models. This modelling approach allows for direct testing of whether the pacing curve differs between conditions, rather than focusing on isolated segments of the trial (Bossi et al., 2025). This is better aligned with pacing as a continuous, time‐evolving process, allowing the pacing profile to be characterised as a function of time while appropriately accounting for the repeated‐measures structure of the data.

Using this approach, no differences were observed in the modelled pacing curve between conditions, indicating neither Competition_BSL_ nor augmented feedback conditions altered how participants distributed effort over time. This suggests that any performance effects of the experimental manipulations occurred without changes in the temporal structure of pacing. Participants maintained a consistent pacing profile across all trials, even when overall performance improved. Comparisons with previous studies are complicated by important methodological differences. Many earlier investigations (Ansdell et al., 2018; Corbett et al., 2012; Ducrocq et al., 2017; Jones et al., 2016a, 2016b; Shei et al., 2016) have examined pacing using point‐by‐point comparisons of discrete segments and absolute power outputs, emphasising localised fluctuations that may be less suited to capturing changes in the overall distribution of effort. Because power output during self‐paced exercise is inherently variable, segment‐based analyses can identify significant differences at isolated time points that do not necessarily reflect meaningful alterations in whole‐task pacing behaviour.

A factor likely contributing to the similar pacing across conditions relates to the time‐based nature of the time trial. As highlighted by Abbiss et al. (2016), time‐based and distance‐based time trials differ in how changes in effort are rewarded. In flat distance‐based trials, increasing power output might directly reduce completion time, providing an immediate incentive to alter pacing. In contrast, during time‐based trials the exercise duration is fixed, and increases in power output do not reduce task duration. Under such conditions, there may be little regulatory or motivational incentive to modify pacing, particularly in familiar time trial protocols where expectations of sustainable effort are well developed. In our study, this lack of immediate ‘reward’ for changing effort distribution might have further reinforced the adoption of similar pacing across conditions.

### 4.4 Limitations

The findings reported in this study should be interpreted considering certain limitations. First, the sample size (*n* = 12) was relatively small. Although the repeated‐measures design reduces between‐participant variability by using each participant as their own control, the precision of some between‐condition estimates was limited, as reflected by the width of confidence intervals. Accordingly, the absence of statistically significant differences between certain conditions should not be interpreted as definitive evidence of no effect. Second, our design involved head‐to‐head competition (1 versus 1) performed in a laboratory. Real‐world mass‐start cycling events involve multiple opponents of varying abilities, creating dynamic drafting opportunities, tactical positioning and psychological responses distinct from those elicited in a 1 versus 1 format. A further limitation is that the study design did not permit full randomisation of time trial order. While the Deception and Accurate conditions were presented in a randomised, counterbalanced order, the preceding sessions necessarily followed a pre‐established order. This design was required to establish a competitive baseline against which the augmented feedback conditions could be compared, but it may have introduced order effects that cannot be disregarded. Additionally, although a deceptive intervention was employed, no formal manipulation check was included to directly assess participants’ beliefs in the experimental procedures. Expectancy differences between conditions and interpretations relating to belief should be made with appropriate caution. Finally, relevant psychological constructs were not directly measured, which limits our ability to fully establish the mechanisms underlying the observed changes (or lack of). Future research should assess constructs such as goal orientation, self‐efficacy and expectancy, measured during exercise alongside physiological markers, to better capture transient psychological states that may shape exercise regulation.

### 4.5 Conclusion

Our study suggests that the effect of head‐to‐head cycling competition on performance is contingent on how the competitive setting is framed. Performance improved relative to Baseline when participants competed against an opponent riding at 2% higher power outputs and the information was disclosed (Accurate). This improvement was accompanied by higher sRPE, likely reflecting greater voluntary engagement with the task. In contrast, no differences between Baseline and Competition_BSL_ or Deception were observed. Pacing remained similar across all conditions, indicating that performance improvements during Accurate occurred without changes in effort distribution.

From an applied perspective, our findings suggest that how competitive tasks are framed might influence performance regulation, even in the absence of substantial physiological perturbations. However, such considerations should be viewed as contextual refinements rather than performance‐enhancing strategies, and their practical relevance likely depends on the competitive setting and athletes’ characteristics.

## AUTHOR CONTRIBUTIONS

Guilherme Matta, Andrew Edwards, Bart Roelands, Florentina Hettinga and Philip Hurst contributed to the conception and design of the study. Guilherme Matta conducted data collection, performed the data analysis, and drafted the manuscript. Guilherme Matta prepared tables and figures. Guilherme Matta, Andrew Edwards, Bart Roelands, Florentina Hettinga and Philip Hurst interpreted the data. Guilherme Matta, Andrew Edwards, Bart Roelands, Florentina Hettinga and Philip Hurst revised and edited the final version of the manuscript. All authors have read and approved the final version of this manuscript and agree to be accountable for all aspects of the work in ensuring that questions related to the accuracy or integrity of any part of the work are appropriately investigated and resolved. All persons designated as authors qualify for authorship, and all those who qualify for authorship are listed.

## CONFLICT OF INTEREST

None declared.

## Data Availability

The dataset supporting the findings of this study is available from the corresponding author upon reasonable request.
